# DICOM-MIABIS integration model for biobanks: a use case of the EU PRIMAGE project

**DOI:** 10.1186/s41747-021-00214-4

**Published:** 2021-05-12

**Authors:** Camilla Scapicchio, Michela Gabelloni, Sara Maria Forte, Leonor Cerdá Alberich, Lorenzo Faggioni, Rita Borgheresi, Paola Erba, Fabiola Paiar, Luis Marti-Bonmati, Emanuele Neri

**Affiliations:** 1 Department of Translational Research on New Technologies in Medicine and Surgery, University of Pisa, Pisa, Italy; 2 Biomedical Imaging Research Group (GIBI230), La Fe Health Research Institute, Valencia, Spain; 3 Medical Imaging Department, La Fe University and Polytechnic Hospital & Biomedical Imaging Research Group (GIBI230), La Fe University and Polytechnic Hospital and Health Research Institute, Valencia, Spain

**Keywords:** Biological specimen banks, Database management systems, Neuroblastoma, Picture archiving and communication system, Radiology

## Abstract

PRIMAGE is a European Commission-financed project dealing with medical imaging and artificial intelligence aiming to create an imaging biobank in oncology. The project includes a task dedicated to the interoperability between imaging and standard biobanks. We aim at linking Digital imaging and Communications in Medicine (DICOM) metadata to the Minimum Information About BIobank data Sharing (MIABIS) standard of biobanking. A very first integration model based on the fusion of the two existing standards, MIABIS and DICOM, has been developed. The fundamental method was that of expanding the MIABIS core to the imaging field, adding DICOM metadata derived from CT scans of 18 paediatric patients with neuroblastoma. The model was developed with the relational database management system Structured Query Language. The integration data model has been built as an Entity Relationship Diagram, commonly used to organise data within databases. Five additional entities have been linked to the “Image Collection” subcategory in order to include the imaging metadata more specific to the particular type of data: Body Part Examined, Modality Information, Dataset Type, Image Analysis, and Registration Parameters. The model is a starting point for the expansion of MIABIS with further DICOM metadata, enabling the inclusion of imaging data in biorepositories.

## Key points

- The availability of imaging biobanks supports tumour decision-making in precision medicine.
- An integration model linking the Minimum Information About BIobank data Sharing and Digital imaging and Communications in Medicine standards has been developed.
- It enables the inclusion of imaging and biomarker data into standard biobanks.

## Background

The availability of biobanks and biorepositories for the research community allows the access to multiple types of data and is the basis for big data analytics in the medical domain [1]. The term biobanking refers to the process by which massive collections of biological material and associated information are collected for research use [2]. Biobanks are defined as repositories for the storage and retrieval of samples of bodily fluids or tissues and accompanying data of the related subject, supporting contemporary research such as genomics and personalised medicine [3, 4].

Digital innovation in the healthcare systems is evolving toward a data-driven science, making high-quality data and information exchange essential in healthcare. It is therefore clear that advances are required in data management to facilitate the development of platforms for more effective usage of large volumes of data, and biobanks play a central role in this scenario [5, 6].

The European Biobanking and BioMolecular resources Research Infrastructure (BBMRI-ERIC) has been created with the aim to harmonise biobanks across Europe and sustain a network of research [7]. The European Society of Radiology (ESR) started a collaboration with BBMRI-ERIC in 2014, recognising the importance of integrating imaging and “omics” data.^1^ All biobanks are indexed in the BBMRI-ERIC Directory from which researchers across Europe can seek retrieval of samples/data, or collect/host services for their samples/data [8, 9].

To integrate different biobanks, the research infrastructure uses a standard for data description named Minimum Information About BIobank data Sharing (MIABIS) 2.0 Core, as a data model standard to describe samples and sample donors. The MIABIS is a recommendation about what information should be stored in biobank to facilitate the exchange of samples information and data. MIABIS represents the standard for the interoperability between biobanks [10, 11].^2^

To date, biobanks are mainly focused on data describing fluid or tissue samples, and very few in BBMRI-ERIC network include image collections [12]. A novel field of research is that of imaging biobanks [13], defined as “organized databases of medical image collections associated with imaging biomarkers” [9]. Imaging biobanks store image collections, just as standard biobanks store biological samples, and donors of images can be described in the same way as donors of samples. Most imaging biobanks focus on the collection of cancer-related data and oncologic imaging biomarkers [14, 15].

A recent challenge is that of reliably connecting the available imaging biobanks to tissue biobanks to explore imaging biomarkers. In fact, the integration of molecular and imaging data is needed for a radiogenomic approach to the patient in a personalised medicine setting [16]. Linkage and integration of existing image data repositories as well as the connection between imaging biobanks and traditional biobanks is the key factor to develop and validate new imaging biomarkers, as well as to improve the general understanding of their biological significance [17].

It is therefore necessary to identify a model of interoperability that describes medical imaging data into the traditional biobanks. To reach this goal a specific task of interoperability has been designed by the PRedictive In-silico Multiscale Analytics to support cancer personalised diaGnosis and prognosis, Empowered by imaging biomarkers (PRIMAGE) project [18, 19]. PRIMAGE is a European Commission-financed project dealing with medical imaging and artificial intelligence aiming to create an imaging biobank in oncology. As part of the interoperability task, the aim of this study was to expand the MIABIS standard with DICOM metadata and provide a model of integration of imaging data and biobank samples.

## Project overview

Here follows a theoretical overview of the method adopted to develop the metadata model that allows the description of imaging data and facilitate interoperability between imaging biomarkers and the traditional biobanks.

To build this model of interoperability among heterogeneous data, the existing formats and ontologies for image and data description, the MIABIS and the DICOM, were considered as standard of reference [20–22]. The MIABIS is the standard that describes the biological samples [10, 11].

**Fig. 1:**
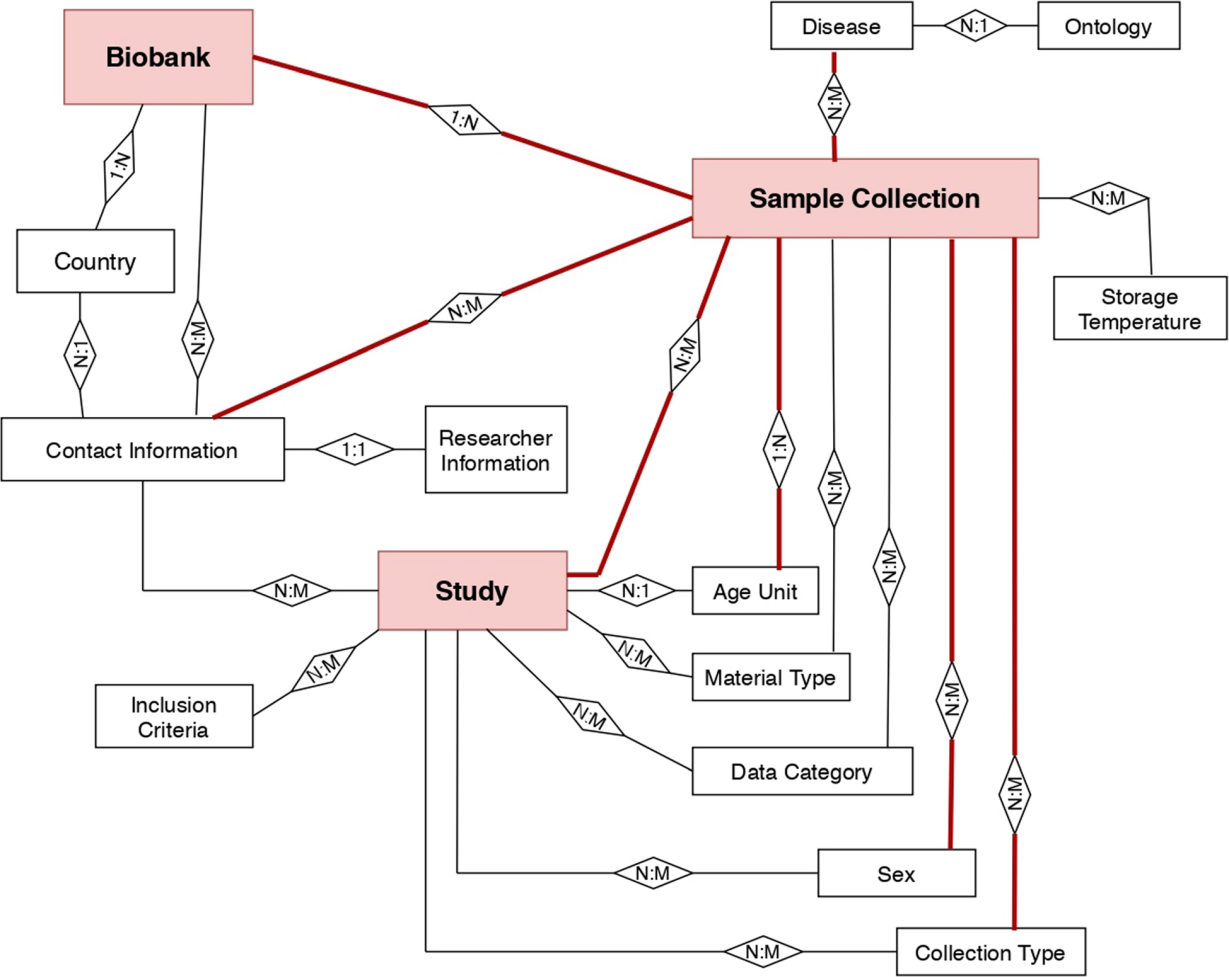
Conceptual schema of the MIABIS core. The conceptual schema and the logical foundation of the MIABIS core used to summarise the data organisation. In the red boxes, the three main categories biobank, study, and sample collection are represented, with the relationships between them and the sub-entities linked to them. The relationships highlighted in red are the ones that have been maintained in the new integration model but linked to “Collection” in place of “Sample Collection.” The abbreviations “1:1,” “1:N,” and “N:M” define the type of relationship in the relational database design. The one-to-one (1:1) relationship exists when zero or one instance of entity A can be associated with zero or one instance of entity B, and vice-versa. The one-to-many (1:N) relationship exists when, for one instance of entity A, there exists zero, one, or many instances of entity B; but for one instance of entity B, there exists zero or one instance of entity A. A many-to-many (M:N) relationship exists when, for one instance of entity A, there exists zero, one, or many instances of entity B, and vice-versa

In the MIABIS core, there are three main well defined components on which the other entities depend: biobank, samples collection, and study (Fig. 1) [10]. MIABIS has a modular structure that allows to add components in a flexible way, which makes the core applicable for a variety of use cases domains of biobanking and research studies. It is possible to attach additional components to the core in order to describe particular subdomains, by merely introducing auxiliary entities in the Entity-Relationship Diagram (ERD) representing the data model.

As it is well known in the imaging domain, DICOM is the standard for the visualisation and sharing of medical images and associated information [23]. In clinical practice, a medical image is a DICOM file. It includes a header in addition to the actual image [24, 25]. All the information stored in the header is cataloged in groups of elements, called “DICOM tags.” Real-world entities (*e.g*., images, procedures, or interpretation reports) are represented in the DICOM semantic data model by templates of attributes or data elements, and each tag identifies an attribute [23, 26].

Therefore, the integration data model here proposed is based on the attempt to create a linkage between the MIABIS format and the DICOM format to represent imaging biomarkers of the PRIMAGE biobank into a network of traditional biobanks.

## Study protocol

The fundamental method used to create the model is based on a fusion between the two existing formats, DICOM and MIABIS standards, mainly describing imaging collections and sample tissue data collections respectively.

The integration data model has been built as an Entity Relationship Diagram (ERD), commonly used to organise data within databases. It is the logical data scheme, which describes the logical organisation and structure of data contained in the database, in a formal language supported by a database management system (DBMS). In a relational database, the scheme defines the given attributes of each table/entity and the relationships between tables. The steps followed to create this model and build the diagram are explained in more detail in the following.

The strategy has been to maintain the fundamental core of the MIABIS data model as a starting point. The conceptual schema and the logical foundation of the ERD describing the MIABIS core are represented in Fig. 1, where the three main categories: biobank, study, and sample collection and the relationships between them and sub-entities have been drawn. In terms of database implementation, the new integration model has been built starting from the MIABIS Entity Relationship Diagram (ERD) coming from the conceptual schema. The tables representing the three main entities with their attributes and the sub-entities linked to them through specific relationships, as represented in the MIABIS conceptual schema (Fig. 1), have been used as a starting point and left almost unchanged in the new extended model, without too many alterations. The MIABIS 2.0 core is modularised and flexible; this means that it is possible to attach additional components to it. We took advantage of this property, *i.e*., we precisely added new components to the core to obtain the new model applicable to the use case of imaging data.

The step necessary to perform the extension of this starting model to the imaging field has been the replacement of the “Sample Collection” entity with a more generic macro category called “Collection,” which includes multiple types of data represented by several sub-categories added to this macro category, *i.e*., “Image Collection,” “Sample Tissue Collection,” “Clinical Variable Collection,” “Molecular Biomarker Collection,” and “Software/Source Code” entities. This is the fundamental step that enables a merge among different types of collections.

The extension of the model to the imaging data has been then done by expanding the “Image Collection” sub-category in two main parts. On the one hand, there is the part regarding the imaging metadata more specific to the particular type of acquired data and based on the DICOM tags. On the other hand, there is a part dependent on the process of image analysis conducted to extract imaging biomarkers and radiomic data. In fact, since the core content of imaging biobanks not only exists out of images but also of any other data (biomarkers) that may be extracted from images through computational analysis, in addition to the classes which represent the information related to imaging data and based on DICOM tags, another part of the model has been developed with other classes describing imaging biomarkers and radiomic data.

At the time of the development of this first model, the imaging data had not yet been uploaded to the PRIMAGE platform. Thus, the model development was not based on how a PRIMAGE imaging biobank sample looks like. Instead, we used the metadata of eighteen DICOM files from cases collected by our Hospital network as examples. In particular, we visualised the DICOM tags of some anonymised multislice CT studies, obtained in paediatric patients with neuroblastoma, available in the PACS system of the Pisa University Hospital, just to perform a preliminary study on which DICOM metadata are worth using for the description of these kind of imaging data. The DICOM tags have been easily visualised using the *Horos* DICOM viewer integrated in the hospital PACS system. The methodology followed to build up this model included also the close collaboration with the other partners of the project and the different professional figures involved in it. Within the PRIMAGE project, researchers at La Fe University and Polytechnic Hospital and Health Research Institute in Valencia developed the radiomics process to extract the imaging biomarkers from neuroblastoma imaging data. Consequently, for the classes of the model describing imaging biomarkers and radiomic data, we referred to the image analysis process performed by this partner. As the project is ongoing, the analysis is described in an internal document, so far available only to the project partners. In this description of the radiomics process, we identified the main elements that can be translated as attributes to properly represent the data coming from the radiomics analysis in the model.

## Software tools

The diagram has been created using the Structured Query Language (MySQL) software [27]. In particular, the MySQL Workbench, which is a graphical tool for working with MySQL servers and databases, has been used to design the database model [28]. This tool enables the graphical creation and manipulation of a model establishing relationships between the tables, where each table represents a single entity. Once defined the ERD, through this tool, it will also be possible to generate the corresponding SQL script.

## Model description

**Fig. 2:**
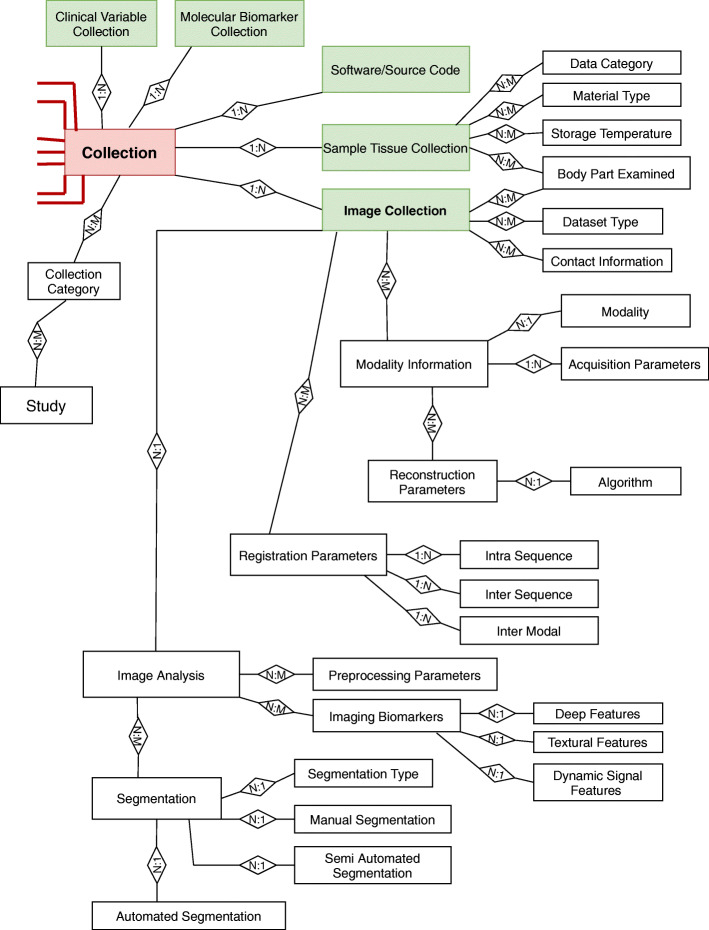
Conceptual schema of the expansion of the model to image collection. The conceptual schema and the logical foundation of the expansion to image collections. The “Collection” entity replaces the “Sample Collection” entity in the conceptual schema of the MIABIS core. The red links are the same ones that connect the “Sample Collection” table to the other tables represented in Fig. 1, with the only difference that the more generic “Collection” replaces “Sample Collection.” The boxes in green are the ones representing the new sub-entities

The conceptual schema of the expansion is represented in Fig. 2, where the red relationships are exactly the same as the ones in the “Sample Collection” entity in MIABIS conceptual schema in Fig. 1, with the “Collection” entity being in place of the “Sample Collection” entity. The “Sample Tissue Collection” in this new schema corresponds to the same entity of the replaced “Sample Collection” in Fig. 1.

**Fig. 3:**
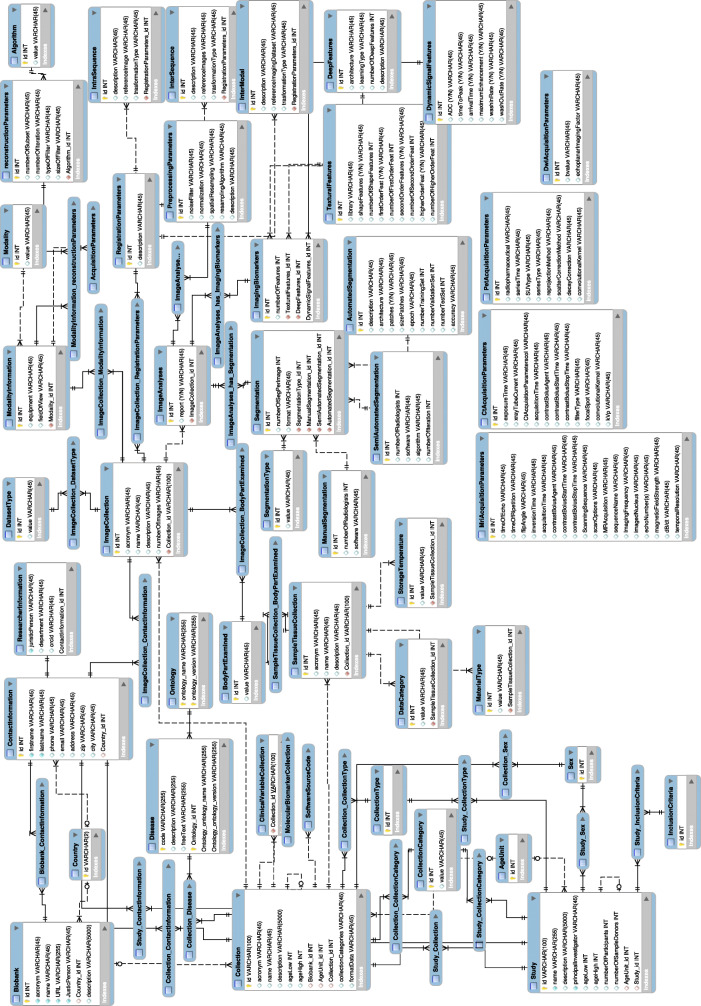
Entity relationship diagram (ERD) of the proposed integration model. The final entity relationship diagram manually created with MySQL Workbench software and representing the data model proposed to integrate imaging data into standard biobanks, in which the imaging part is linked to the MIABIS core. This is the structural diagram typically used in informatics to visualise the database design idea, with the tables representing the entities, with a set of attributes describing each entity, and the links representing the relationships among the entities. It is just used as visual support of the structure we want to use for data description, but it gives an idea of the complexity of the model

The complete ERD of the resulting model with the imaging part linked to the MIABIS core is represented in Fig. 3. This is the structural diagram manually drawn in MySQL Workbench and typically used in informatics to visualise the database design idea, with the tables representing the entities, with a set of attributes describing each entity, and the links representing the relationships among the entities.

**Fig. 4:**
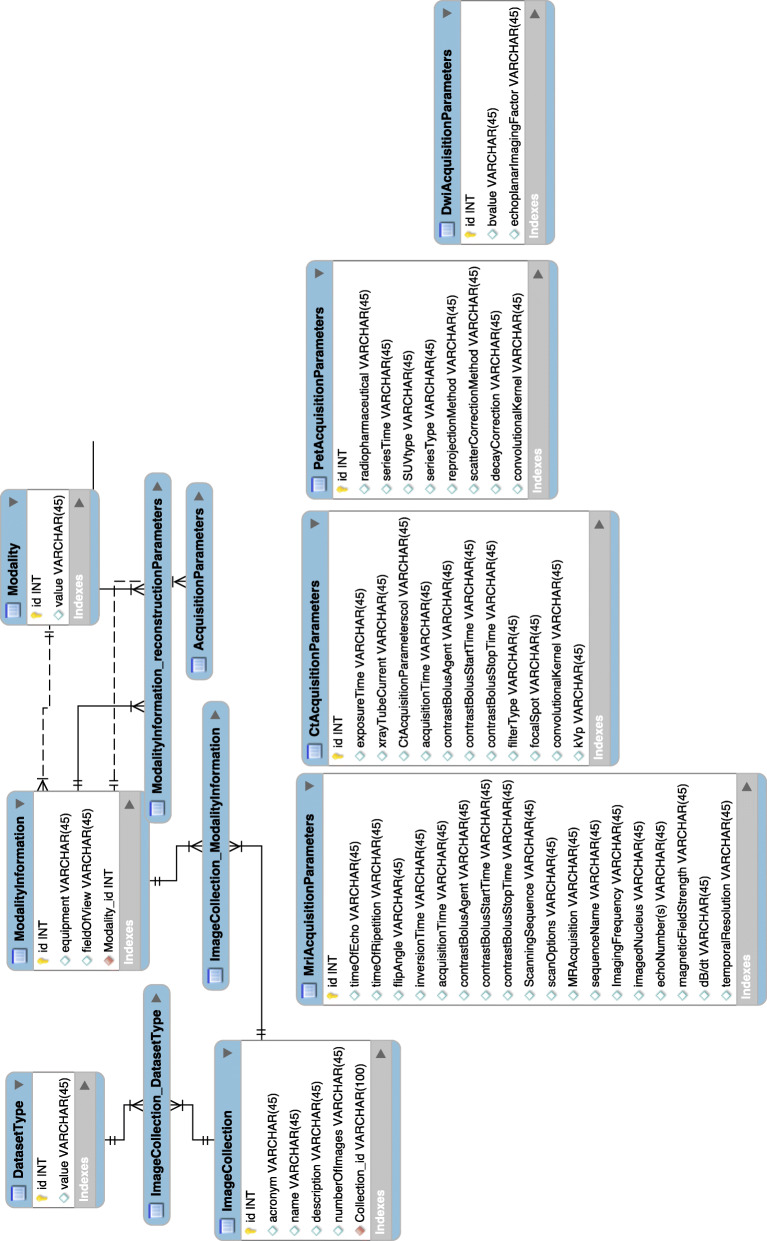
Tables in the model representing the entities based on DICOM tags. A zoom on some tables, *i.e*., some entities (dataset type, modality information, acquisition parameters) used in the model to describe the acquired imaging data, with the attributes based on the most relevant DICOM metadata

**Fig. 5:**
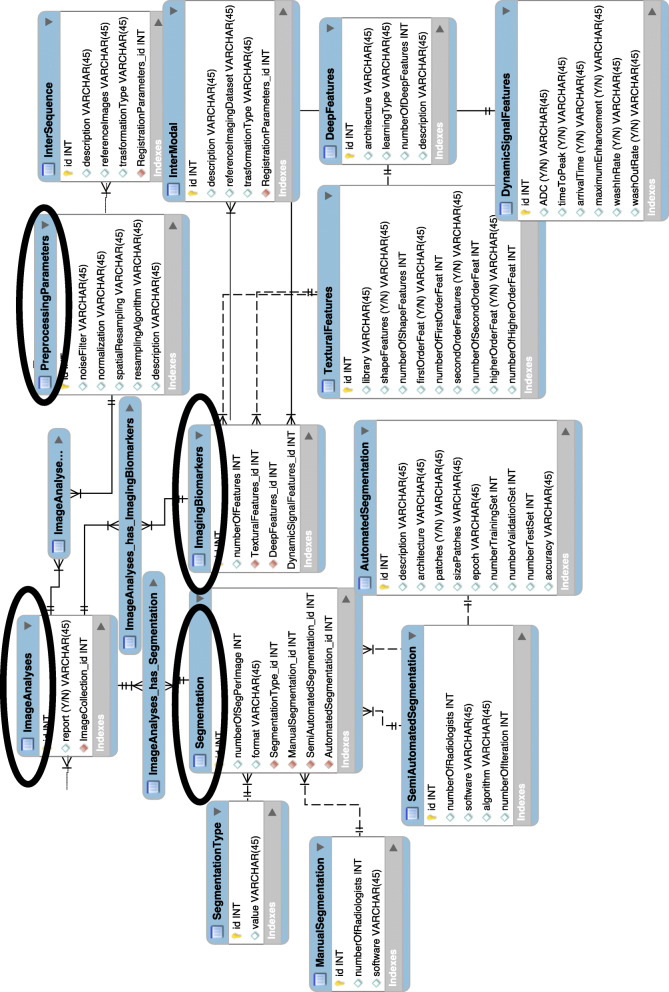
Tables in the model representing the entities based on the image analysis process. A zoom on some tables, *i.e*., some entities (segmentation, imaging biomarkers, preprocessing parameters) used in the model to describe the process of analysis of the acquired images, conducted to extract the imaging biomarkers, with the attributes selected on the basis of the process of radiomics features extraction developed and described by the other partners of the project

This is the model that allows defining a unified view and description of data and will provide access to data themselves (also imaging data, in this case) and to the related clinical information through a simple query processing by researcher users, even though data are heterogeneous. We also split the entire model in Fig. 3 into Figs. 4 and 5 with a zoom on the most relevant parts of the model, the one describing the DICOM metadata and the one describing the process of image analysis for image biomarkers extraction.

In this new extended final model (Fig. 3), based on the schematically simplified expansion represented in Fig. 2, it is evident that among the sub-entities that were in relationship with the “Sample Collection” in the MIABIS ERD, those specifically related to tissue samples, such as storage temperature and material type, have only been linked to the “Sample Tissue Collection” sub-entity. However, the more generic sub-entities, *i.e*., those describing patient’s information, such as age and sex, have been obviously linked to the more generic macro category “Collection.”

Five additional entities are linked to the “Image Collection” sub-category in order to include the imaging metadata more specific to the particular type of data: body part examined, modality information, dataset type, image analysis and registration parameters [23, 29, 30]. This expansion is the fundamental step that enables to extend the MIABIS model, only describing tissue sample data, to the imaging data.

The Body Part Examined entity is based on its respective DICOM tag (0018, 0015) related to DICOM Part 16 which provides a list of 116 terms describing with a text the part of the body examined. It may also be replaced by Anatomic Region Sequence DICOM tag (0008, 2218).

The dataset type entity is likewise based on its corresponding DICOM tag (0008, 0016) related to DICOM Part 16 that provides a list of 120 terms and code values. The Modality Information entity describes the equipment that originally acquired the data used to create the images in the series and is in relationship with modality, acquisition parameters, and reconstruction parameters. Modality is based on the DICOM tag (0008, 0060) related to DICOM Part 03 which provides a list of 54 terms and code values.

With regard to this part of the schema based on the DICOM metadata, only some attributes describing the various entities have been established. In Fig. 4, we show some tables from the entire model in Fig. 3, with the attributes based on the selected DICOM tags. In the future development of the model, a more accurate examination and analysis on the meaningful DICOM tags that must be used as attributes to describe the specific type of imaging data will be further explored.

In the part concerning the modality information, the image analysis, and the registration parameters, the tables representing the entities and the relationships between entities and sub-entities have been established on the basis of the image analysis process and the consequent biomarkers extraction implemented in the PRIMAGE project. In addition, given the description of this image analysis process, the attributes describing these entities have been selected (Fig. 5).

The registration parameters entity is linked to intra-sequence, inter-sequence, and inter-modal sub-entities. The image analysis entity has pre-processing parameters, segmentation, and imaging biomarkers as sub-entities. Segmentation is then divided into three other sub-entities which describe the segmentation modality: manual, semi-automatic, and automatic. Imaging biomarkers is instead divided into the type of extracted features: texture feature, deep features (both related to the radiomics analysis), and dynamic signal features.

The use-case of integration between these two standards, DICOM and MIABIS, is a very first model and it is a starting point for standardisation of imaging data and metadata representation for data sharing.

## Discussion

It is important to remark that the purpose of this work is to provide a theoretical description of the model and, in particular, of the idea behind the construction of the integration. The described software tool was just used as a visual support to the realisation of the model, which still needs to be validated on real data. As soon as the data are available, we will have the model ready to be used to obtain practical results confirming the initial idea here described. The step of data collection is still ongoing, so we did not link the model to concrete data yet, but we built the model on the basis of the information generally known about the data to describe them.

The significant novelty of the DICOM-MIABIS integration model developed in this study is the preparation for interoperability among imaging biobanks and traditional biobanks of the BBMRI network [18].

The proposed model will bring major advancements in facilitating imaging data cross-linking with other traditional wider biorepositories. This will have significant potential to link imaging biomarkers with other biomarkers and clinical data. The inclusion of new imaging biobanks in standard biobanks is crucial to achieve data integration, to access large amounts of high-quality data and, consequently, to foster a personalised medicine setting [31].

A key feature that a biobank must have to be included in BBMRI is to provide access to data and clinical information through a simple query processing. In fact, data from multiple sources can be heterogeneous, *i.e*., with different structure, models, query languages, and semantics. Therefore, data integration, which consists in providing a unified view and access of multiple datasets to users, is fundamental. The described data model, based on the proposed EER diagram, enables to organise the imaging data in PRIMAGE biobank in such a way as to meet this request, integrating imaging data and related information through a unified view [32, 33].

Another aspect that makes relevant the description of this model is that in BBMRI-ERIC Directory only one imaging biobank with CT images of COVID-19 pertaining to the Netherlands node has already been included, and there does not exist a description of the data model used for the data integration [34]. As a result, imaging biobanks and their implementation into the already existing biobanks is a very recent field of research, in the literature, there is no detailed description of a database model that allows imaging data to be integrated into a network of traditional biobanks, which are based on a standard that is only limited to describing tissue samples. The novelty of the present work consists in providing a descriptive model that should offer a possible solution on how to effectively connect imaging and tissue biobanks, giving guidance on how to organise the database to include and add medical images and associated metadata and biomarkers to other types of data.

The model has been constructed referring to the use case of neuroblastoma (NB) and diffuse intrinsic pontine glioma (DIPG) tumours, because imaging and radiomic data in PRIMAGE biobank focuses on these two paediatric cancers and no neuroblastoma or DIPG biobanks already exist. However, the model could also be extended to be adapted to other solid tumours use-cases.

Within the specific framework of the PRIMAGE project, this model will contribute to provide a strategy for embedding PRIMAGE imaging biobanks into wider biobanks networks (*e.g*., BBMRI-ERIC), but, generally, since the advancement of in-silico medicine research is intrinsically linked to the availability of curated databases, a generalisation of such an integration model could play a part in boosting precision medicine in oncology, contributing to easier access to data and their use for AI analysis, which will hopefully provide improved health outcomes in general. In fact, for a personalised assessment of the disease imaging biomarkers alone are not sufficient, and they must be considered in conjunction with other biological data [35]. Consequently, the possibility to access biobanks including different kinds of data, *e.g*., imaging and biological, related to the same patient could be crucial to link images and biomarker data to other omics, such as genomic profiling, metabolomics, proteomics, lab values, and clinical information. In order to create this integration of data, it is extremely important to have a database model as a precisely described tool to efficiently organise the data [17, 36].

**Fig. 6:**
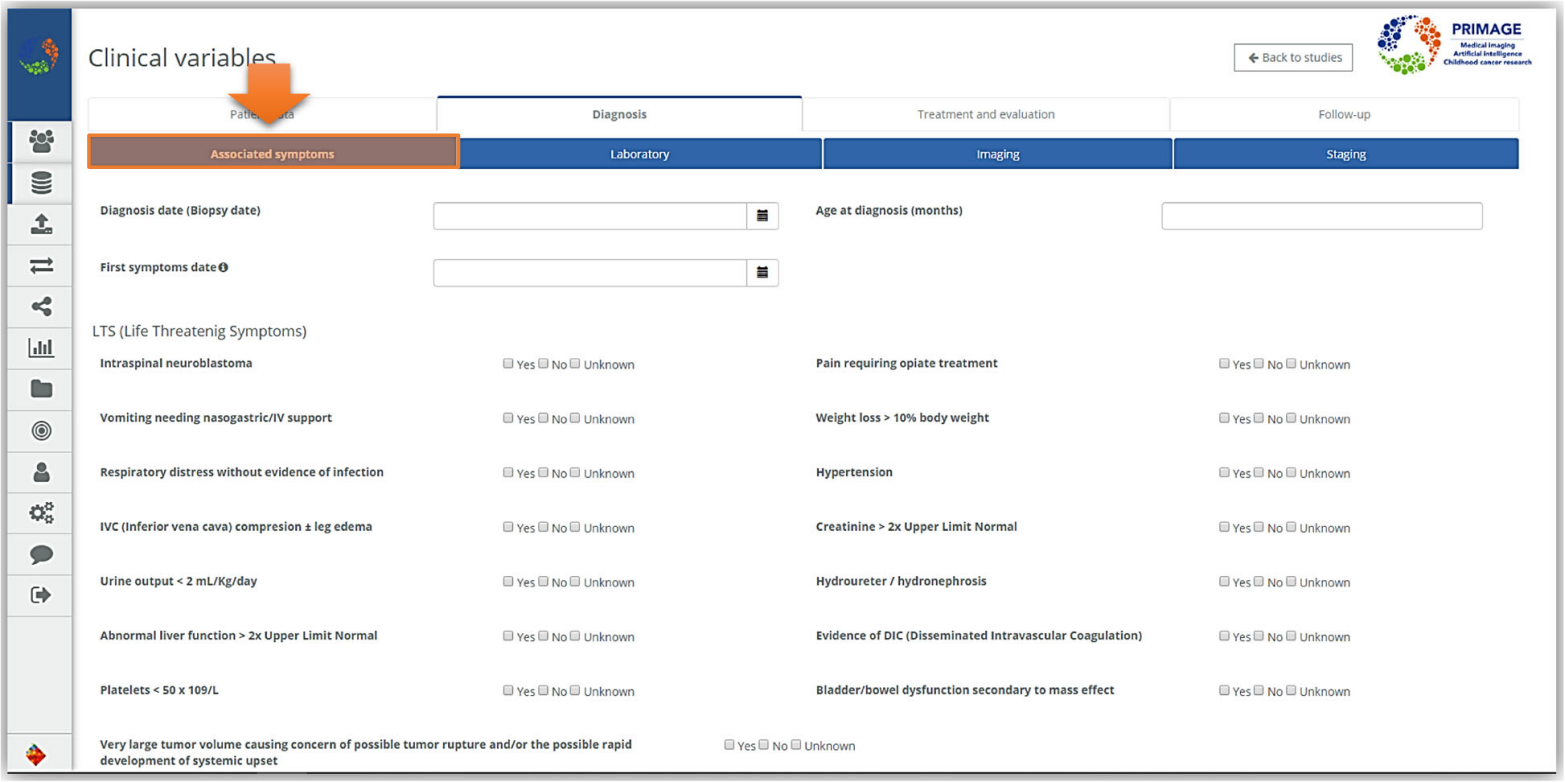
Example of an e-form representing some of the clinical variables related to a PRIMAGE sample. Detailed information about the associated symptoms at the time of diagnosis in the diagnosis section of clinical variables in an e-form. This is an example of how it appears in the PRIMAGE platform

**Fig. 7:**
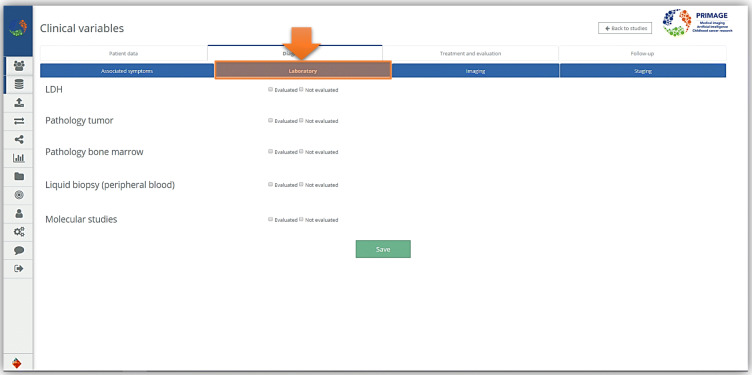
Example of part of an e-form representing some clinical variables related to a PRIMAGE sample. Detailed information about the laboratory tests in the diagnosis section of clinical variables in an e-form. This is an example of how it appears in the PRIMAGE platform

In conclusion, the model built so far and here described is only a first proposal of integration between MIABIS and DICOM formats. This model will be further evolved by extending the descriptions of image collections in order to figure out the necessary information to describe imaging data and define the best way to represent them into biobanks. In fact, as mentioned, this first model has been built relying on the description of the image and radiomics analysis conducted by the institution responsible for the generation of imaging and radiomic data, without having access to the real data. After the development of this proposal model, the first real imaging studies and the related clinical variables have been uploaded to the e-form in two separate projects (NB and DIPG) and are now accessible through the web platform. At the moment a PRIMAGE imaging biobank sample is described in the platform through an e-form containing all the relevant clinical variables (Figs. 6 and 7), while the imaging metadata are simply contained in the imaging metadata part of the separated DICOM file.

Therefore, in a subsequent study, it should be possible to fine-tune this first model by studying which DICOM metadata should be effectively included and which particular imaging biomarkers and radiomic features are worth representing and linking to other kinds of data (*e.g*., clinical), now relying on real uploaded data. After having explored the PRIMAGE platform data storage and having understood how the data and the related clinical variables are organised, it will be possible to further improve the integration model by adding only the attributes that will be considered necessary and the clinical biomarkers assessed as the meaningful ones.

Furthermore, MIABIS Core is currently being updated to version 3.0. The update will include additional extensions which have been identified by BBMRI-ERIC working group. Consequently, a possible future outlook could be that of modifying the integration model to adapt it to this currently being updated version 3.0 of MIABIS core.

The model here theorised is a first attempt to link the MIABIS standard with the DICOM standard, whose final aim is to simplify access to knowledge, improve interoperability, standardisation and data management, and to ensure a harmonised approach to quality assurance of data, enabling an integration of imaging and “omics” data with external resources (other biobanks) and the creation of a structured repository for imaging data in PRIMAGE biobank to describe them to the scientific community.

The innovative aspect and the scientific relevance of this work lie in the possibility of advancement toward reuse of datasets, which is essential to accelerate the uptake of the use of big data in medicine research.

## Data Availability

Project website (https://www.primageproject.eu/)

## Abbreviations

BBMRI-ERIC: European Biobanking and BioMolecular resources Research Infrastructure
DICOM: Digital Imaging and COmmunications in Medicine
DIPG: Diffuse Intrinsic Pontine Glioma
ERD: Entity Relationship Diagram
MIABIS: Minimum Information About BIobank data Sharing
NB: NeuroBlastoma
PRIMAGE: PRedictive In-silico Multiscale Analytics to support cancer personalised diaGnosis and prognosis, Empowered by imaging biomarkers
SQL: Structured Query Language

## Footnotes

1 The last author of this paper has been chair of the Imaging Biobanks working group of the European Society of Radiology.

2 The first (corresponding) author of this paper is also member of the MIABIS working group.
